# Pneumococci detected in sterile body fluids of pediatric patients postpandemic trends from a single center

**DOI:** 10.1017/ash.2026.10314

**Published:** 2026-04-21

**Authors:** Gülnihan Üstündağ, Eda Karadağ Öncel, Aslıhan Şahin, Ayşegül Elvan Tüz, Yıldız Ekemen Keleş, Selin Taşar Karabulut, Nesli Ağralı Eröz, Güliz Doğan, Dilek Yılmaz, Nisel Yılmaz, Gülşen Hasçelik, Mehmet Ceyhan

**Affiliations:** 1 Pediatric Infectious Diseases Clinic, Gaziantep City Hospital, Gaziantep, Türkiye; 2 Department of Pediatric Infectious Diseases, Dokuz Eylul University Faculty of Medicine, Türkiye; 3 Pediatric Infectious Diseases Clinic, Kahramanmaraş Necip Fazıl City Hospital, Kahramanmaraş, Türkiye; 4 Department of Pediatric Infectious Diseases, İzmir City Hospital, Türkiye; 5 Department of Pediatric Infectious Diseases, Health Science University İzmir Çiğli Training and Research Hospital, Türkiye; 6 Department of Pediatric Infectious Diseases, Health Science University Tepecik Training and Research Hospital, İzmir, Türkiye; 7 Microbiology Laboratory, Health Science University Izmir Tepecik Training and Research Hospital, Türkiye; 8 Department of Medical Microbiology, İzmir City Hospital, İzmir, Türkiye; 9 Department of Medical Microbiology, Hacettepe University Faculty Of Medicine, Türkiye; 10 Division of Pediatric Infectious Diseases, Hacettepe University Faculty Of Medicine, Ankara, Türkiye

## Abstract

This study assessed pediatric invasive pneumococcal disease (IPD) post-COVID-19. No cases appeared in the first two pandemic years, but 12 were diagnosed after 2022. Identified serotypes included 3, 6B, 19, and untypeable G. Despite PCV13, infections persist, especially serotype 3, notable for its robust capsule.

## Introduction

After the ominous silence of the very post-coronavirus diseases 2019 (COVID-19) period, respiratory viruses took over their ancient roles again, culprit agents for most respiratory infections. After their appearance, some bacterial infections in childhood that normally cause mild to moderate illnesses manifested as more severe and invasive infections as a result of not encountering various viruses due to the pandemic precautions, which led the immune system of children to remain naïve.^1,2^

*Streptococcus pneumonia*, on the other hand, despite vaccination efforts, has always been causing invasive infections such as pneumonia, meningitis, complicated otitis, and abscesses.^3^ Some serotypes, such as 19F and 3, were especially accused of causing invasive infections due to their unusually robust polysaccharide capsules, although they exist in the pneumococcal conjugate vaccine (PCV) 13.^4^ Various studies have been complemented to determine the serotype distribution of invasive diseases and nasopharyngeal carriage because variations can occur in different settings and vaccine implementation procedures.^5,6^

This study assessed patients with pneumococcal invasive infections, the serotype distribution of these infections, and the patients’ characteristics post-COVID-19 era.

## Methods

### Design

This case series aimed to detect pediatric patients diagnosed with invasive pneumococcal disease (IPD) during the following three years after the COVID-19 pandemic emerged.

### Setting

The patient population consists of patients with IPD who were hospitalized in our tertiary care hospital. Our hospital is a large tertiary-care center located in the third largest city in the country and serves as a referral hospital for surrounding smaller cities. It comprises multiple medical and surgical units, including internal medicine and its subspecialties, providing care for both pediatric and adult patients. Overall, the hospital admits approximately 60,000 patients in the wards annually, while the pediatric emergency department evaluates nearly 180,000 patients each year.

Patients with IPD were followed and treated by our pediatric infectious diseases team, which consists of two professors, one associate professor, pediatric infectious disease fellows, and pediatric registrars.

### Patient and participants

Patients younger than 18 years who were diagnosed with IPD between March 2020 and April 2023 were included. Patients with IPD were defined as those in whom Streptococcus pneumoniae was identified by culture or polymerase chain reaction (PCR) from normally sterile body sites, including blood, cerebrospinal fluid, urine, abscess material, and bronchoalveolar lavage specimens. Cases with a positive pneumococcal result only from multiplex respiratory PCR panels using nasopharyngeal swabs were excluded, as these samples do not represent sterile sites and may reflect nasopharyngeal carriage.

In addition to demographic characteristics of the patients such as gender and age, presence of underlying diseases that facilitate IPD, the length of hospitalization, antibiotic treatment received, antibiotic susceptibility, duration of treatment, body site of pneumococcal growth, pneumococcal serotypes, laboratory and imaging tests, and pneumococcal vaccination status of the patients recorded in the case report form prepared by the researchers.

All data except the vaccination status was accessed from our hospital’s software system, and the pneumococcal vaccination status of the patients was obtained from the electronic health records and vaccination card provided by family physicians to the families. Pneumococci-grown samples were sent for serotyping to the Public Health Laboratory and/or the reference laboratory.

## Results

During the first two years of the pandemic, no patient was detected as infected with pneumococcus. As of the beginning of 2022, among 12 patients who were diagnosed with IPD, seven (58.3%) were female and five (41.7%) were male. The mean age was 61.8 ± 59.2 months. Five of the patients (41.7%) had an underlying facilitating factor. Seven (58.3%) of patients’ isolates were able to be sent for PCR analysis and came back positive. The median length of hospitalization and treatment of the patients was 15 (IQR: 11–27). Only one patient with mastoiditis and cerebral abscess was unvaccinated; two with mastoiditis had an incomplete course with PCV13. The rest of them were vaccinated with a 3-dose schedule of PCV13. Detected serotypes of pneumococci were 3, which was detected two times in different patients, 6B, 19, and unserotypable G. Additional details of patients’ characteristics are shown in Table 1.

**Table 1.** Demographic, clinical and laboratory characteristics of the patients

	Date of admission	Age (months)	Facilitating factors	Diagnosis	Length of hospitalization (days)	Length of treatment (days)	Antibiotic susceptibility test			PCR*	Pneumococcus serogroup	Immunization status
							*Penicillin*	*Cephalosporin*	*Vancomycin*			
Case 1	February 2022	204	None	Cerebral abscess secondary to mastoiditis	82	82	S	S	S	N/A	N/A	Unvaccinated
Case 2	March 2022	108	Nephrotic syndrome	Peritonitis, septicemia	14	14	R	–	S	+	6B	PCV13/3 dose
Case 3	March 2022	7	None	Septicemia, pneumonia	14	14	S	–	S	+	3	PCV13/2 dose
Case 4	April 2022	4	None	Mastoiditis	14	14	S	S	–	N/A	N/A	PCV13/2 dose
Case 5	June 2022	36	Cochlear implantation	Mastoiditis	7	7	S	–	–	+	19	PCV13/3 dose
Case 6	June 2022	48	Acute otitis media	Mastoiditis, brain abscess	28	28	–	–	–	N/A	N/A	PCV13/3 dose
Case 7	July 2022	8	None	Mastoiditis	10	10	–	–	–	+	N/A	PCV13/2 dose
Case 8	October 2022	60	None	Septicemia, pneumonia, lung abscess	54	53	S	–	–	+	3	PCV13/3 dose
Case 9	November 2022	108	Liver transplant, acute otitis media	Liver transplantation mastoiditis, epidural abscess	27	27	–	–	–	+	N/A	PCV13/3 dose
Case 10	January 2023	84	ALL	Septicemia	10	10	S	–	S	N/A	N/A	PCV13/3 dose
Case 11	January 2023	2	None	Mastoiditis	16	16	S	S	S	N/A	G, unserotypable	PCV13/1 dose
Case 12	March 2023	72	None	Mastoiditis	22	22	S	S	S	+	N/A	PCV13/3 dose

*In some cases, sample volume was insufficient, and at times PCR testing was temporarily unavailable at our hospital; therefore, certain specimens could not be analyzed by PCR.

## Discussion

Consistent with emerging global reports, we observed a distinct absence of IPD cases during the initial years of the pandemic, aligning with data from regions like England and Catalonia, where lockdowns and non-pharmaceutical interventions resulted in a marked reduction in IPD incidence. As social restrictions were lifted, however, IPD cases re-emerged, particularly among younger populations, suggesting that limited exposure to pathogens may have led to an “immunity debt,” increasing susceptibility to respiratory and bacterial infections once normal social interactions resumed.^7,8^ In our investigation, no IPD cases were seen during the first year of the pandemic. Three of the 12 patients were observed before April 2022, while the remaining cases, or 75% of the total, emerged after this date. This finding is especially significant because our country’s ministry of health abolished the requirement to wear a mask in public on April 27, 2022. Within the two-year period following the pandemic, only three cases were observed, whereas nine cases were identified during the final year of the study. In comparison, our previously published prepandemic case series in the same hospital setting reported eight patients within a one-year period.^9^ Notably, in both case series, at least one patient was infected with serotype 3 despite having completed full immunization with PCV13. Breakthrough invasive pneumococcal disease can occur despite vaccination because immune protection differs between serotypes. Some vaccine serotypes, especially serotype 3, are known to have lower or less consistent vaccine effectiveness.^10^ In addition, certain vaccine serotypes may continue to circulate in the community over time, leading to ongoing exposure and occasional invasive disease even in vaccinated individuals.^11^ When the clinical manifestations of IPD were examined in our pre and postpandemic case series, meningitis was more frequently observed in the prepandemic period, whereas mastoiditis emerged as the predominant presentation in the postpandemic period. However, robust scientific conclusions cannot be drawn due to the small sample size of the study.

In our current case series, the detected pneumococcal serotypes included serotypes 3, 6B, 19, and an untypable serotype G. Notably, serotype 3, which is included in PCV13, was detected in multiple cases and is recognized for its robust polysaccharide capsule, which may contribute to its invasive potential despite vaccination coverage. The persistence of serotype 3 in invasive cases is consistent with findings from England, where serotype 3 remains a prominent cause of IPD postpandemic due to its limited response to PCV13-induced immunity.^7^ Serotype 3 was found to be most frequent in our study as well similar to Silva-Costa’s study in which serotype 3 was also the culprit agent for vaccine breakthrough.^12^ Serotype 19F, another serotype associated with invasive potential, also appeared in our study, reflecting findings from other regions that report increased IPD cases associated with these serotypes, particularly as respiratory viruses re-emerged and co-facilitated bacterial invasions.^13,14^ In a prepandemic pneumococcal serotype study, serotype 19F was seen predominantly among IPD cases.^9^

The clinical profile of our patients revealed that 41.7% had underlying conditions that could predispose them to IPD, with prolonged hospitalization duration and severe presentations such as mastoiditis and cerebral abscesses observed. This observation aligns with literature indicating more severe manifestations of typically mild infections as a result of postpandemic immune naivety, particularly in children whose immune systems had less pathogen exposure during lockdown periods.^9,15^

The fact that only one patient with IPD was unvaccinated while others had completed or partially completed the PCV13 schedule underlines the ongoing role of vaccination in reducing IPD incidence and severity. However, despite the introduction of PCV13, invasive infections continue to occur with serotypes already included in PCV13. The persistent appearance of serotypes like 3 and 19F highlights a potential limitation in current PCV13 coverage, especially for serotypes with strong capsules that evade vaccine immunity. Studies suggest that while PCV13 continues to offer valuable protection, it is crucial to maintain high vaccine coverage and introduce broader-spectrum vaccines for preventing IPD.^8,14,16^

There are several limitations in our study, including the method of collecting vaccination information. Ideally, vaccination status should be obtained from electronic health records rather than vaccination cards. However, vaccination cards were used as proof because some older children did not have electronic records, as documentation practices were manual at that time. Other important limitations include the small sample size and the single-center design of the study. Due to these factors, the findings may not be sufficient for reliable interpretation, and further multicenter studies with larger sample sizes are needed. Additionally, serotyping could not be performed for all patients. As this was a retrospective design, patients were treated during the acute phase of their illness, and determining pneumococcal serotypes was not a primary objective; the focus was on clinical management of pneumococcal infections. Consequently, serotype distribution could not be fully assessed, which represents an additional limitation of the study.

In conclusion, our study supports growing evidence that the COVID-19 pandemic has impacted IPD epidemiology, with the re-emergence of respiratory pathogens contributing to increased disease burden. Ongoing surveillance of serotype dynamics, coupled with efforts to sustain high vaccination coverage, particularly with newly available broader-spectrum vaccines such as PCV15 and PCV20, will be essential in controlling IPD in the evolving postpandemic landscape.^17^
